# Parabrachial nucleus Vglut2 expressing neurons projection to the extended amygdala involved in the regulation of wakefulness during sevoflurane anesthesia in mice

**DOI:** 10.1111/cns.70001

**Published:** 2024-08-18

**Authors:** Yan Yan, Yingfu Jiao, Enpeng Liang, Xiaolu Lei, Nan Zhang, Shimao Xu, Lin Zhang, Jiedong Wang, Tianyuan Luo, Jie Yuan, Chengdong Yuan, Hao Yang, Hongxin Dong, Tian Yu, Weifeng Yu

**Affiliations:** ^1^ Department of Anesthesiology, Renji Hospital Shanghai Jiao Tong University School of Medicine Shanghai China; ^2^ Key Laboratory of Anesthesiology (Shanghai Jiao Tong University) Ministry of Education Shanghai China; ^3^ Key Laboratory of Anesthesia and Organ Protection of Ministry of Education (In Cultivation) Zunyi Medical University Zunyi China; ^4^ Guizhou Key Laboratory of Anesthesia and Organ Protection Affiliated Hospital of Zunyi Medical University Zunyi China; ^5^ Guizhou Key Laboratory of Brain Science Zunyi Medical University Zunyi China; ^6^ Department of Anesthesiology Affiliated Hospital of Zunyi Medical University Zunyi China; ^7^ Department of Pain Affiliated Hospital of Zunyi Medical University Zunyi China; ^8^ Department of Anesthesiology The Second Affiliated Hospital of Zunyi Medical University Zunyi China; ^9^ Department of Anesthesiology, Shanghai Pulmonary Hospital Tongji University School of Medicine Shanghai China; ^10^ Department of Psychiatry and Behavioral Sciences Northwestern University Feinberg School of Medicine Chicago Illinois USA

**Keywords:** consciousness, general anesthesia, parabrachial nucleus, the extended amygdala, wakefulness

## Abstract

**Aims:**

The parabrachial nucleus (PBN) promotes wakefulness states under general anesthesia. Recent studies have shown that glutamatergic neurons within the PBN play a crucial role in facilitating emergence from anesthesia. Our previous study indicates that vesicular glutamate transporter 2 (vglut2) expression neurons of the PBN extend into the extended amygdala (EA). However, the modulation of PBN^vglut2^‐EA in general anesthesia remains poorly understood. This study aims to investigate the role of PBN^vglut2^‐EA in alterations of consciousness during sevoflurane anesthesia.

**Methods:**

We first validated vglut2‐expressing neuron projections from the PBN to the EA using anterograde tracing. Then, we conducted immunofluorescence staining of c‐Fos to investigate the role of the EA involved in the regulation of consciousness during sevoflurane anesthesia. After, we performed calcium fiber photometry recordings to determine the changes in PBN^vglut2^‐EA activity. Lastly, we modulated PBN^vglut2^‐EA activity under sevoflurane anesthesia using optogenetics, and electroencephalogram (EEG) was recorded during specific optogenetic modulation.

**Results:**

The expression of vglut2 in PBN neurons projected to the EA, and c‐Fos expression in the EA was significantly reduced during sevoflurane anesthesia. Fiber photometry revealed that activity in the PBN^vglut2^‐EA pathway was suppressed during anesthesia induction but restored upon awakening. Optogenetic activation of the PBN^vglut2^‐EA delayed the induction of anesthesia. Meanwhile, EEG recordings showed significantly decreased δ oscillations and increased β and γ oscillations compared to the EYFP group. Furthermore, optogenetic activation of the PBN^vglut2^‐EA resulted in an acceleration of awakening from anesthesia, accompanied by decreased δ oscillations on EEG recordings. Optogenetic inhibition of PBN^vglut2^‐EA accelerated anesthesia induction. Surprisingly, we found a sex‐specific regulation of PBN^vglut2^‐EA in this study. The activity of PBN^vglut2^‐EA was lower in males during the induction of anesthesia and decreased more rapidly during sevoflurane anesthesia compared to females. Photoactivation of the PBN^vglut2^‐EA reduced the sensitivity of males to sevoflurane, showing more pronounced wakefulness behavior and EEG changes than females.

**Conclusions:**

PBN^vglut2^‐EA is involved in the promotion of wakefulness under sevoflurane anesthesia. Furthermore, PBN^vglut2^‐EA shows sex differences in the changes of consciousness induced by sevoflurane anesthesia.

## Graphical abstract

The PBN^vglut2^‐EA is involved in the modulation of awakening during sevoflurane anesthesia. The PBN^vglut2^‐EA exhibited sexually dimorphic activity patterns under general anesthesia. The activation of the PBN^vglut2^‐EA reduced sensitivity to sevoflurane in males and resulted in enhanced arousal states and EEG changes.

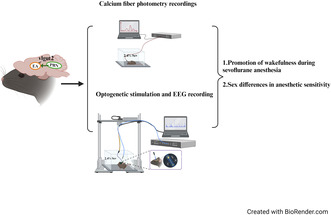

## 1 INTRODUCTION

The reversible loss of consciousness during general anesthesia provides valuable insights into the mechanisms underlying alterations in consciousness.^1^, ^2^, ^3^, ^4^, ^5^ Researchers have found similarities between the mechanisms of general anesthesia and sleep–wake neural circuits,^6^ leading to a better understanding of the impact of relevant brain regions on these processes. Recent studies suggest that the effects of general anesthesia are not limited to a single brain region.^7^, ^8^ Therefore, it is imperative to investigate the structural connectivity between the brain regions involved in regulating general anesthesia.

The parabrachial nucleus (PBN), an essential part of the cerebral bridge, is a major component of the ascending reticular activating system.^9^ The PBN contains mostly glutamatergic neurons that project widely to various brain regions, including the cerebral cortex, thalamus, hypothalamus, and basal forebrain.^10^ These projections are implicated in the regulation of essential physiological functions, including the control of sleep–wake cycles, pain perception, and sensory transmission.^11^ The PBN is a pivotal nucleus in the maintenance of wakefulness, with its associated sleep–wake circuit playing a critical role in this process. The activation of PBN neurons using the chemogenetic method in animals resulted in a prolonged period of sustained wakefulness.^11^ Previous research has indicated that glutamatergic neurons within the medial PBN interact with the basal forebrain and lateral hypothalamus to facilitate behavioral and cortical wakefulness.^12^ The specific knockout of the glutamatergic neuronal marker gene vglut2 in the PBN led to a decreased duration of wakefulness, accompanied by an increase in EEG slow waves.^13^ Notably, our preliminary findings demonstrated that activation of PBN glutamatergic neurons during isoflurane and propofol anesthesia accelerated the emergence time.^14^ Additionally, activation of glutamatergic neurons in the PBN can prolong the induction period of sevoflurane anesthesia and significantly reduce the emergence time.^15^ These studies indicate that the activity of glutamatergic neurons in the PBN is essential for influencing behavior and EEG patterns during general anesthesia. Furthermore, PBN activation induced neuronal activity in various brain regions, including the prefrontal cortex, lateral hypothalamus, and basal forebrain.^14^ This suggests that the PBN glutamatergic projection may be involved in regulating sevoflurane‐induced alterations in consciousness, but the specific mechanisms remain unknown.

The extended amygdala (EA), a complex structure of the basal forebrain, comprises the central amygdala (CeA), the nucleus accumbens shell (NAc), and the bed nucleus of the stria terminalis (BNST), which receive projections from PBN glutamatergic neurons.^16^ The CeA mediates a variety of behaviors, including sleep–wake behavior and defensive responses.^17^, ^18^ The majority of neurons in the CeA are associated with sleep–wake behavior. Studies have shown that the firing of CeA neurons can be detected by electrophysiological tests, with these neurons firing slowly during nonrapid eye movement (NREM) sleep and increasing during rapid eye movement (REM) sleep or wakefulness.^19^ The administration of tetrodotoxin into the CeA resulted in a significant reduction of REM sleep and a notable decrease in wake duration.^20^ The BNST is another crucial nucleus that plays a role in regulating sleep–wake cycles. Optogenetic activation of GABA neurons in the BNST leads to an immediate transition from NREM to wakefulness in mice.^21^ Recently, there has been growing evidence to suggest that the BNST plays a role in regulating consciousness during anesthesia.^22^ During sevoflurane anesthesia, a temporary activation of the PVT‐BNST can effectively induce behavioral alertness and decrease the depth of anesthesia.^23^ The study of the functionality of PBN‐associated neural networks during general anesthesia could provide a more comprehensive understanding of the mechanism of general anesthesia. Given that PBN glutamatergic neurons project to the EA, it is essential to explore the PBN‐EA interaction in the modulation of wakefulness under general anesthesia.

## 2 MATERIALS AND METHODS

### 2.1 Animals

C57BL/6J and vGLUT2‐IRES‐Cre mice were housed in a standard SPF laboratory animal room with a regular 12/12 h light/dark cycle (light onset at 7:00 a.m.; temperature: 22 ± 2°C; relative humidity: 50 ± 5%). The vGLUT2‐IRES‐Cre mouse strain, which expresses Cre recombinase under the control of the vglut2 gene promoter, was provided by Prof. Wei Shen (ShanghaiTech University, Shanghai, China). C57BL/6J mice were provided by Chongqing Tengxin Technology Co., Ltd. (Chongqing, China). They were given free access to food and water. A total of 72 mice (males and females) were equally distributed to each group. All mice weighed 20–30 g (8–12 weeks old) in this study. The experimental procedures in this study were approved by the Animal Care and Use Committees of Zunyi Medical University. All experimental procedures followed the Guide for the Care and Use of Laboratory Animals in China (No. 14924, 2001).

### 2.2 Stereotaxic surgery and viral delivery

The vGLUT2‐Cre mice were anesthetized in an induction chamber. They were placed on a stereotactic apparatus (RWD Life Sciences, Shenzhen, China) and anesthetized during the procedure using 1.4% isoflurane with oxygen at a rate of 1 L/min. Local anesthesia was provided by subcutaneous injection of 2% lidocaine. Then, the cranial surface was exposed. The bregma and lambda lines were used to align the horizontal position of the skull. A small window with a diameter of 0.6 mm was opened at the site for cre‐dependent virus (Brain‐VTA, Wuhan, China) injection and fiber implantation. The cre‐dependent virus was injected using a calibrated glass micropipette connected to a microsyringe pump. Assays were performed after 21 days of virus expression. All animals were individually housed and given sufficient time to fully recover before commencing the formal experiment.

To provide clarification on the projection of PBN_vglut2_ to EA, we injected AAV‐Ef1a‐DIO‐EGFP‐WPRE‐hGH (Brain‐VTA, Wuhan, China) virus into the PBN region (anterior–posterior [AP]: −5.20 mm, medial‐lateral [ML]: ± 1.4 mm, and dorsal‐ventral [DV]: −3.5 mm) following the coordinates provided by Paxinos and Franklin's atlas (2013) in vGLUT2‐Cre mice. After the injection, the micropipette was left in place for 10 min and then withdrawn.

Fiber photometry was utilized to observe the dynamic alterations in the PBN^vglut2^‐EA. vGLUT2‐Cre mice were unilaterally injected with rAAV‐hSyn‐DIO‐axon‐GCaMP8m (120 nL/site at a speed of 30 nL/min; Brain‐VTA, Wuhan, China) into the PBN region (anterior–posterior [AP]: −5.20 mm, medial‐lateral [ML]: ± 1.4 mm, and dorsal‐ventral [DV]: −3.5 mm). After the injection, the micropipette was left in place for 10 min and then withdrawn. The ceramic optical fiber cannula (diameter: 200 μm; NA: 0.37, Inper Technology Co., Ltd) was unilaterally implanted over the EA (anterior–posterior [AP]: −0.45 mm, medial‐lateral [ML]: ±1.5 mm, dorsal‐ventral [DV]: −4.7 mm).

Utilizing an optogenetic approach, the activation/inhibition of PBN^vglut2^‐EA was achieved by bilaterally injecting Cre‐dependent viruses (AAV‐EF1a‐DIO‐ChR2‐EYFP, AAV‐EF1a‐DIO‐eNpHR3.0‐EYFP, Brain‐VTA, Wuhan, China) and eYFP (AAV‐EF1a‐DIO‐EYFP) (120 nL/site at a speed of 30 nL/min, Brain‐VTA, Wuhan, China) into the PBN at specific coordinates (anterior–posterior [AP]: −5.20 mm, medial‐lateral [ML]: ± 1.4 mm, and dorsal‐ventral [DV]: −3.5 mm). The mice were bilaterally implanted with optical fibers (diameter: 200 μm; NA: 0.37, Inper Technology Co., Ltd) in the EA (anterior–posterior [AP]: −0.45 mm, medial‐lateral [ML]: ±1.5 mm, dorsal‐ventral [DV]: −4.6 mm) and electroencephalography (EEG). The recording electrodes were small stainless‐steel screws fixed in the skull above the cerebellum. The electrodes and fibers were firmly attached to the skull with dental cement.

### 2.3 Evaluation of induction and emergence times

The standard indicators for loss of the righting reflex (LORR) and recovery of the righting reflex (RORR) have been utilized to evaluate the induction and emergence of general anesthesia in mice. The mice were placed in an anesthetic induction chamber (10 × 20 × 15 cm) and anesthetized with 2.4% sevoflurane and oxygen (1 L/min). LORR is defined as the mice maintaining the supine position for over 30 s, while RORR signifies that the mice returned to the prone position after the cessation of anesthesia. The temperature within the induction chamber was consistently regulated at 37.5°C during the entire anesthesia process.

### 2.4 Calcium fiber photometry recordings

Fiber photometry recording utilized modulated 470 and 410 nm LEDs from Inper Technology Co., Ltd. The power output of the 470 nm LED varied between 20 and 40 μW, while the 410 nm LED ranged from 10 to 20 μW. The optical path was coupled to a 200 mm NA:0.37 fiber optic patch cord and then attached to a ceramic fiber implanted in each mouse (Figure 2A). Stimulating the brain region expressing GaMP8m with a 470 nm LED produced a calcium‐dependent fluorescent signal, whereas stimulating the same region with a 410 nm LED produced a calcium‐independent fluorescent signal. The functional connectivity of PBN^vglut2^‐EA terminals was assessed by monitoring GaMP8m fluorescence changes, indicative of neuronal calcium activity in the PBN^vglut2^‐EA during alterations in anesthetic consciousness.

The mice were placed in the induction chamber and monitored for 5 min during spontaneous activity, and a 50 s preanesthesia period was recorded as the baseline. Subsequently, the mice were anesthetized with 2.4% sevoflurane. They were exposed to 2.4% sevoflurane for 15 min until the onset of LORR. Data collection concluded 5 min after the recovery of RORR (Figure 2A). Data were collected for 100 s, and analysis was conducted at the time points when sevoflurane inhalation started and ceased.

The ratio of fluorescence changes was calculated by processing the raw data using software (Inper Ltd., China) and a custom MATLAB code (Inper Ltd., China). The data were divided into segments in accordance with the behavioral events observed in each individual experiment. The 410 nm signal was adjusted using the principle of least squares regression to reduce the discrepancy between the 410 and 470 nm signals. Subsequently, the scaled 410 nm signal was subtracted from the 470 nm signal, yielding the final corrected signal value. The fluorescence of the calcium signal activity was calculated using the following formula for Δ*F*/*F*: Δ*F*/*F* = (*F*(*t*) − *F* *0*)/*F0*, where *F* is the test fluorescence signal and *F0* is the average baseline fluorescence value.

### 2.5 In vivo optogenetic stimulation during sevoflurane anesthesia

In this study, we employed an optogenetic technique to modulate the PBN^vglut2^‐EA in order to investigate its involvement in sevoflurane anesthesia. AAV‐EF1a‐DIO‐ChR2‐EYFP or AAV‐EF1a‐DIO‐eNpHR3.0‐EYFP was bilaterally injected into the PBN (Figure 3B). Implanted fibers were used to activate or inhibit PBN^vglut2^‐EA in the bilateral EA. An optogenetic system from Inper Technology Co., Ltd was connected to the fibers to emit either blue or yellow light. Blue light (470 nm, 5 mW, 5 ms pulse, 20 Hz) or yellow light (589 nm, 5 mW, 5 ms pulse, 20 Hz) was used for photoactivation/photoinhibition during the LORR and RORR phases. Simultaneously, behavioral and EEG alterations in the test subjects were recorded (Figure 3A).

### 2.6 EEG recording and spectral analysis

The EEG signals were acquired using a multichannel signal acquisition system (Apolo, Bio‐Signal Technologies, USA) and connected to an amplifier (A‐M Systems, USA). The EEG recording process included: 5 min of preadministration, maintenance of inhalation of 2.4% sevoflurane anesthesia for 20 min after LORR, and 5 min after RORR (Figure 3A). The signals were filtered between 0.1 and 300 Hz, bandpass filtered from 1 to 60 Hz, and a spectrogram was generated. Power spectrum analysis for optogenetic experiments was conducted during the induction and emergence periods of sevoflurane anesthesia. The data were filtered to capture δ (1–4 Hz), θ (4–8 Hz), α (8–12 Hz), β (12–25 Hz), and γ (25–60 Hz) band recordings using Spike 2 software (Cambridge Electronic Design). Subsequently, the power in each band was normalized against the total power from 1 to 60 Hz, as per established methodologies.

### 2.7 Immunofluorescence staining

Neuronal depolarization triggers the expression of the immediate early gene c‐Fos and its nuclear product, c‐Fos protein. Accordingly, c‐Fos staining is a widely utilized method for identifying activated neurons during various physiological states in the central nervous system, including sleep and wakefulness. This staining technique is also valuable for investigating the impact of general anesthetics on neuronal nuclear activity. The C57BL/6J mice were randomly assigned to the sevoflurane anesthesia, anesthesia recovery, and wakefulness groups to examine the activity of EA neurons during anesthesia. The mice were placed in an induction chamber and exposed to a stable concentration of 2.4% sevoflurane for inhalation to induce anesthesia. Following this, they were monitored for 2 h after the onset of LORR. The sevoflurane anesthetized group was sampled immediately, while the recovery group was monitored for an additional 2 h after recovery of the RORR. The wakefulness group was sampled directly without any treatment.

Mice were anesthetized with 1.4% isoflurane followed by perfusion with phosphate‐buffered saline (PBS) and 4% paraformaldehyde (PFA). Brains were fixed in 4% PFA at 4°C overnight and then dehydrated in 30% sucrose at 4°C. Brains were sliced into 30 μm coronal slices using a cryostat (CM1950; Leica, Germany). These slices were rinsed with PBS three times for 5 min each. The sections were then blocked with 0.3% Triton X‐100 and 10% donkey serum in PBS for 2 h at room temperature. The sections were then incubated with the primary c‐Fos antibody (1:1000, Abcam, ab222699) diluted in PBST with 5% donkey serum at 4°C overnight. After three rinses with PBS for 5 min each, brain slices were incubated with the secondary antibody (1:1000, Alexa Fluor 589, Abcam, ab150065) diluted in PBST with 5% donkey serum for 2 h at room temperature. Cell nuclei were counterstained with DAPI (Life Technologies, USA). Imaging was performed using an Olympus BX63 virtual microscopy system.

### 2.8 Statistical analysis

Statistical analysis was performed using Prism 9.0 (GraphPad Software, San Diego, CA, USA). Data are presented as mean ± SEM. Prior to analysis, normality and homogeneity of variance of all datasets were assessed using the Kolmogorov–Smirnov and Brown–Forsythe tests. Student's *t*‐test was employed to compare c‐Fos counts and LORR and RORR durations. Event‐related differences in neuronal calcium signal changes were analyzed using a Student's *t*‐test. Sex differences in calcium signal changes and EEG results were assessed through a two‐way ANOVA with Bonferroni post hoc tests for multiple comparisons. Statistical significance was set at *p < 0.05, **p < 0.01, ***p < 0.001, ****p < 0.0001.

## 3 RESULTS

### 3.1 Anatomical and molecular characterization of PBN^vglut2^ ‐EA

**FIGURE 1:**
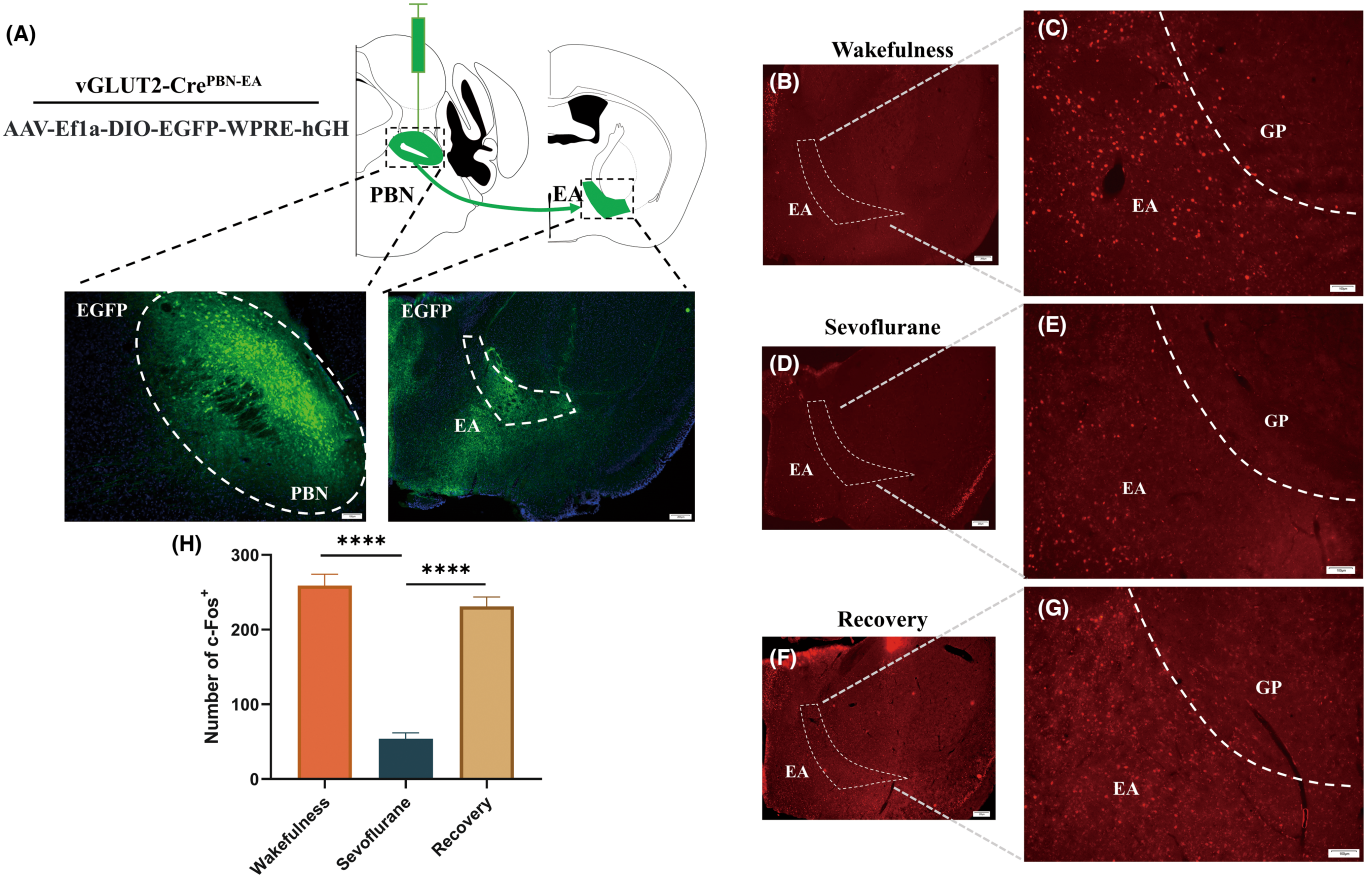
Anatomical and molecular characterization of PBN^vglut2^‐EA. (A) Schematic of viral injection and a representative image depicting expression in PBN_Vglut2_ and its terminals in the EA. (B, C) The expression of c‐Fos in the EA during wakefulness (E: Scale bar, 200 μm, F: Scale bar, 100 μm). (D, E) The expression of c‐Fos in the EA under sevoflurane anesthesia (A: Scale bar, 200 μm, B: Scale bar, 100 μm). (F, G) The expression of c‐Fos in the EA after recovery from sevoflurane anesthesia (C: Scale bar, 200 μm, D: Scale bar, 100 μm). (H) Quantification and comparison of c‐Fos expression in the wakefulness, sevoflurane, and recovery groups. The results are shown as the mean ± SEM, *n* = 6 for each group, *p < 0.05, **p < 0.01, ***p < 0.001, ****p < 0.0001.

In order to elucidate the projection of the downstream brain region of PBN_vglut2_, we injected the anterograde adeno‐associated viruses (AAV‐Ef1a‐DIO‐EGFP‐WPRE‐hGH) into the PBN of vGLUT2‐Cre mice. Anterograde tracing revealed glutamatergic PBN neurons that innervate the EA (Figure 1A). To further substantiate the EA neuronal activity during sevoflurane anesthesia, c‐Fos protein immunofluorescence staining was employed. C57BL/6J mice were randomly divided into the sevoflurane anesthesia, anesthesia recovery, and wakefulness groups. The sevoflurane anesthesia group (Figure 1D,E) showed a significant decrease in c‐Fos expression after anesthesia compared to the wakefulness group (Figure 1B,C) (sevoflurane: 53.83 ± 7.96 vs. wakefulness: 258.8 ± 15.41, *p* < 0.0001, *n* = 6 per group) (Figure 1H), whereas c‐Fos expression was significantly increased after recovery of consciousness (Figure 1F,G) (recovery: 231.2 ± 12.49 vs. sevoflurane: 53.83 ± 7.96, *p* < 0.0001, *n* = 6 per group) (Figure 1H). These findings indicate that neurons in the EA may have been inhibited during sevoflurane anesthesia and activated during the recovery process from sevoflurane anesthesia.

### 3.2 Changes in PBN^vglut2^ ‐EA activity during sevoflurane anesthesia

**FIGURE 2:**
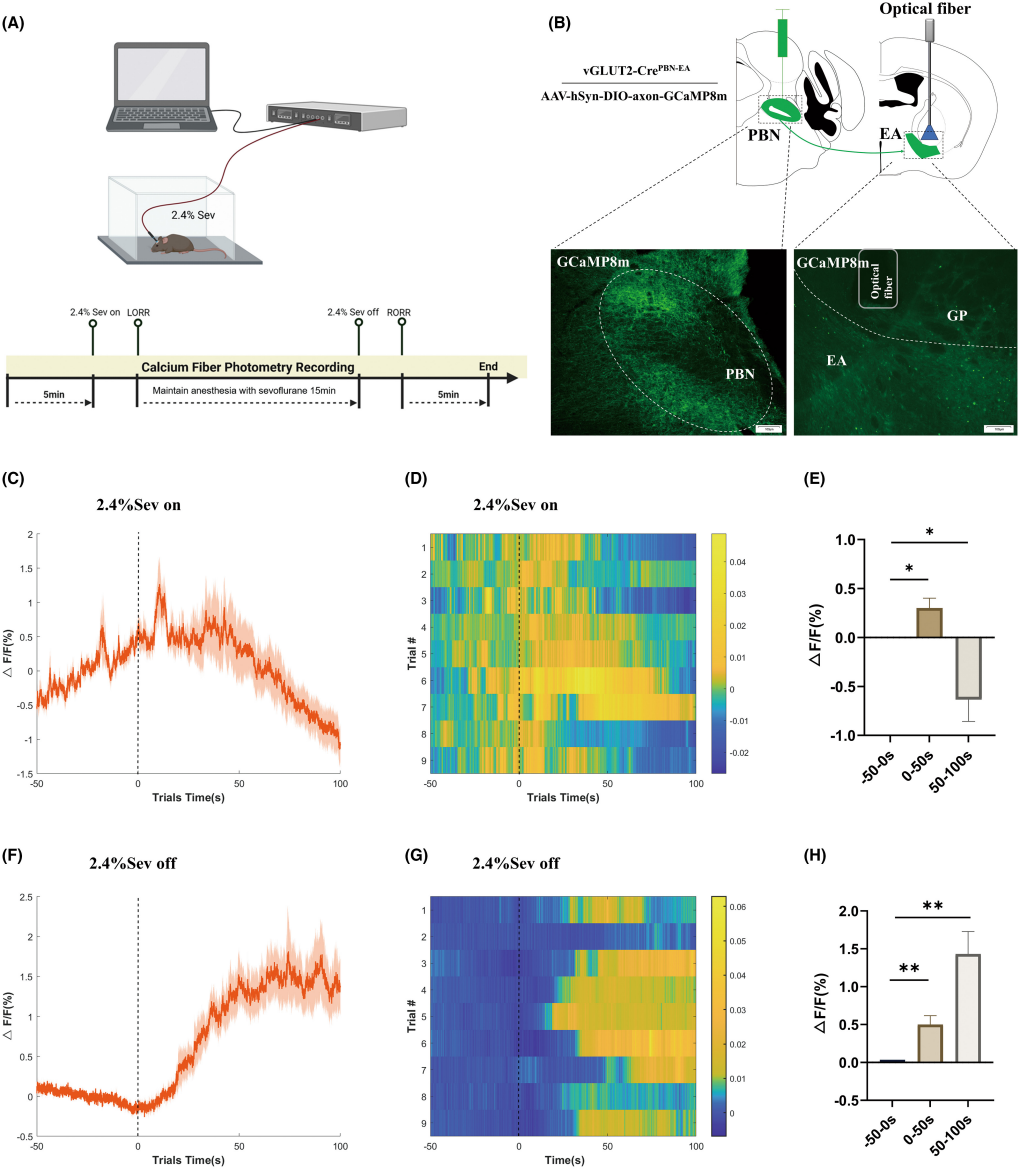
Calcium activity of PBN^vglut2^‐EA terminals during sevoflurane anesthesia. (A) Schematic of the in vivo fiber photometry recording procedure (Created with BioRender.com). (B) Representative GCaMP8m expression in the PBN^vglut2^‐EA and optical fiber location of a vGLUT2‐Cre mice. Scale bars, 100 μm. (C) Plot of sevoflurane anesthesia‐induced event‐related curves, with the red line representing the mean calcium activity intensity of PBN^vglut2^‐EA terminals and shading indicating SEM. (D) Heatmap of PBN^vglut2^‐EA terminals calcium activity during the induction of sevoflurane anesthesia (Δ*F*/*F* indicates fluorescence intensity). Experimentally composed calcium activity changes during anesthesia were shown, with dashed lines indicating the time point at which anesthesia was initiated. (E) Quantitative analysis of the changes in the PBN^vglut2^‐EA terminals calcium activity during the induction of sevoflurane anesthesia showed a significant decrease in calcium activity intensity after anesthesia (phase II: 0.3014 ± 0.1001 vs. phase I: 0.0001059 ± 0.00014, *p =* 0.0168, phase III: −0.6350 ± 0.2224 vs. phase I: 0.0001059 ± 0.00014, *p =* 0.0213). (F) PBN^vglut2^‐EA terminals activity in relation to emergence events during sevoflurane anesthesia. (G) Heatmap of the PBN^vglut2^‐EA terminals calcium activity during sevoflurane emergence (Δ*F/F* indicates fluorescence intensity). (H) Quantitative analysis of the changes in PBN^vglut2^‐EA terminals calcium activity during emergence from sevoflurane anesthesia showed a significant increase in PBN^vglut2^‐EA terminals calcium activity intensity (phase II: 0.5015 ± 0.1161 vs. phase I: −0.00003465 ± 0.00002965, *p =* 0.0025, phase III: 1.432 ± 0.2964 vs. phase I: −0.00003465 ± 0.00002965, *p =* 0.0013). The results are presented as the mean ± SEM, *n* = 9 in each group, *p < 0.05, **p < 0.01.

To assess changes in PBN^vglut2^‐EA activity during sevoflurane anesthesia, we employed calcium fiber photometry to monitor calcium‐mediated fluorescence (Figure 2A). AAV‐hSyn‐DIO‐axon‐GCaMP8m was targeted to the PBN of vGLUT2‐Cre mice, with an optical fiber positioned above the EA to measure PBN‐EA glutamatergic terminals calcium activity (Figure 2B). The vGLUT2‐Cre mice were anesthetized with 2.4% sevoflurane. The onset of anesthesia inhalation was taken as the time reference, and anesthesia induction was divided into three phases: baseline (phase I: −50–0 s), anesthesia induction (phase II: 0–50 s), and early anesthesia (phase III: 50–100 s). During the induction of anesthesia, a transient increase in GCaMP activity was observed in phase II (phase II: 0.3014 ± 0.1001 vs. phase I: 0.0001059 ± 0.00014, *p =* 0.0168, *n* = 9 per group). This was followed by a significant decrease in early anesthesia (phase III: −0.6350 ± 0.2224 vs. phase I: 0.0001059 ± 0.00014, *p =* 0.0213, *n* = 9 per group) (Figure 2C–E).

The anesthesia recovery period was also divided into three phases: baseline (phase I: −50–0 s), emergence (phase II: 0–50 s), and early recovery (phase III: 50–100 s), with the cessation of continuous anesthesia used as the time reference point. The vGLUT2‐Cre mice were exposed to 2.4% sevoflurane anesthesia for 15 min. Following the cessation of anesthesia, significantly elevated activity was observed during both phase II and phase III of the anesthesia recovery period, in comparison to phase I (phase II: 0.5015 ± 0.1161 vs. phase I: −0.00003465 ± 0.00002965, *p =* 0.0025, phase III: 1.432 ± 0.2964 vs. phase I: −0.00003465 ± 0.00002965, *p =* 0.0013, *n* = 9 per group) (Figure 2F–H). These results suggest that PBN^vglut2^‐EA is involved in the modulation of sevoflurane anesthesia.

### 3.3 Optical activation of PBN^vglut2^ ‐EA delayed induction and facilitated recovery during sevoflurane anesthesia

**FIGURE 3:**
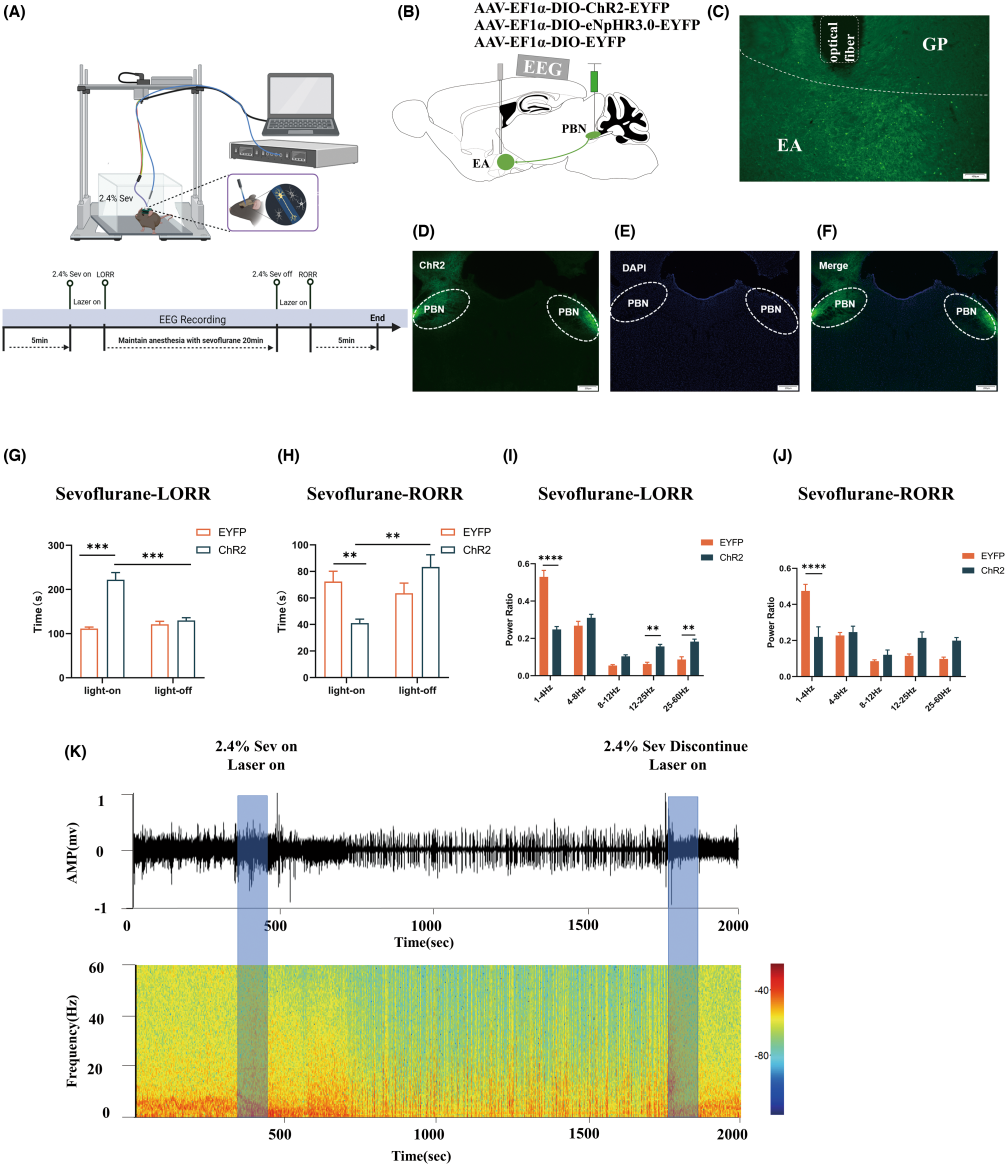
Optogenetic activation of PBN^vglut2^‐EA delayed induction and enhanced recovery under sevoflurane anesthesia. (A) Optogenetics approach operating procedure during sevoflurane anesthesia (Created with BioRender.com). (B) Schematic of the excitatory optogenetic virus (ChR2) injection into the PBN in vGLUT2‐Cre mice. (C) Schematic of the optical fiber location in the EA diagram. (D‐F) Schematic of PBN optogenetic virus (ChR2) expression. (G) Photoactivated PBN^vglut2^‐EA showed a significantly increased LORR (ChR2‐light‐on: 222.1 s ± 16.34 s vs. EYFP‐light‐on: 111.3 s ± 3.529 s, *p =* 0.0002; ChR2‐light‐on vs. ChR2‐off: 129.9 s ± 6.262 s, *p =* 0.0005). (H) Photoactivated PBN^vglut2^‐EA shows a significantly decreased RORR time (ChR2‐light‐on: 41.06 s ± 2.944 s vs. EYFP‐light‐on: 72.38 s ± 7.783 s, *p* = 0.0045, ChR2‐light‐on vs. ChR2‐light‐off: 83.38 s ± 9.178 s, *p =* 0.0020). (I) Photoactivation during sevoflurane induction causes reduced δ oscillations (0.2476 ± 0.01578 vs. 0.5291 ± 0.03507, *p* < 0.0001), and increased β (0.1561 ± 0.0117 vs. 0.06247 ± 0.0095, *p =* 0.0017) and γ oscillations (0.1820 ± 0.0138 vs. 0.08686 ± 0.01426, *p =* 0.0014). (J) During sevoflurane recovery, optical activation of the PBN^vglut2^‐EA caused a significant decrease in δ oscillations (0.2195 ± 0.05625 vs. 0.4746 ± 0.0366, *p* < 0.0001). (K) Photoactivated PBN^vglut2^‐EA EEG spectra and heatmap. The results are presented as the mean ± SEM, *n* = 8 in each group, *p < 0.05, **p < 0.01, ***p < 0.001, ****p < 0.0001.

We targeted the excitatory optogenetic virus (ChR2) (Figure 3B) to the bilaterally PBN (Figure 3D–F) of vGLUT2‐Cre mice, with optical fibers positioned above the EA (Figure 3C) for photostimulation (470 nm, 5 mW, 5 ms pulse, 20 Hz) of PBN‐EA glutamatergic terminals. EEG was used to record the instantaneous changes in consciousness during sevoflurane anesthesia (Figure 3A).

Activation of ChR2 in the PBN^vglut2^‐EA during induction of sevoflurane anesthesia significantly prolonged the LORR time (ChR2‐light‐on: 222.1 s ± 16.34 s vs. EYFP‐light‐on: 111.3 s ± 3.529 s, *p =* 0.0002; ChR2‐light‐on vs. ChR2‐off: 129.9 s ± 6.262 s, *p =* 0.0005, *n* = 8 per group) (Figure 3G). After cessation of continuous sevoflurane inhalation, the RORR time was significantly shorter following simultaneous photoactivation of the PBN^vglut2^‐EA (ChR2‐light‐on: 41.06 s ± 2.944 s vs. EYFP‐light‐on: 72.38 s ± 7.783 s, *p =* 0.0045, ChR2‐light‐on vs. ChR2‐light‐off: 83.38 s ± 9.178 s, *p =* 0.0020, *n* = 8 per group) (Figure 3H).

The EEG recording was performed throughout the entire anesthesia procedure (Figure 3K). The ChR2 group showed a significantly decreased relative power of δ oscillation compared to the EYFP group (0.2476 ± 0.01578 vs. 0.5291 ± 0.03507, *p* < 0.0001, *n* = 8 per group). Additionally, the relative powers of β (0.1561 ± 0.0117 vs. 0.06247 ± 0.0095, *p =* 0.0017) and γ oscillations (0.1820 ± 0.0138 vs. 0.08686 ± 0.01426, *p =* 0.0014) were significantly increased (Figure 3I). Photoactivation caused a significant decrease in δ oscillations at the RORR in the ChR2 group (0.2195 ± 0.05625 vs. 0.4746 ± 0.0366, *p* < 0.0001, *n* = 8 per group) (Figure 3J). These results suggest that activation of the PBN^vglut2^‐EA during sevoflurane anesthesia results in a prolonged induction time to facilitate anesthesia emergence and this process is reflected by corresponding cortical EEG changes.

### 3.4 Optical inhibition of the PBN^vglut2^ ‐EA accelerates the induction of sevoflurane anesthesia

**FIGURE 4:**
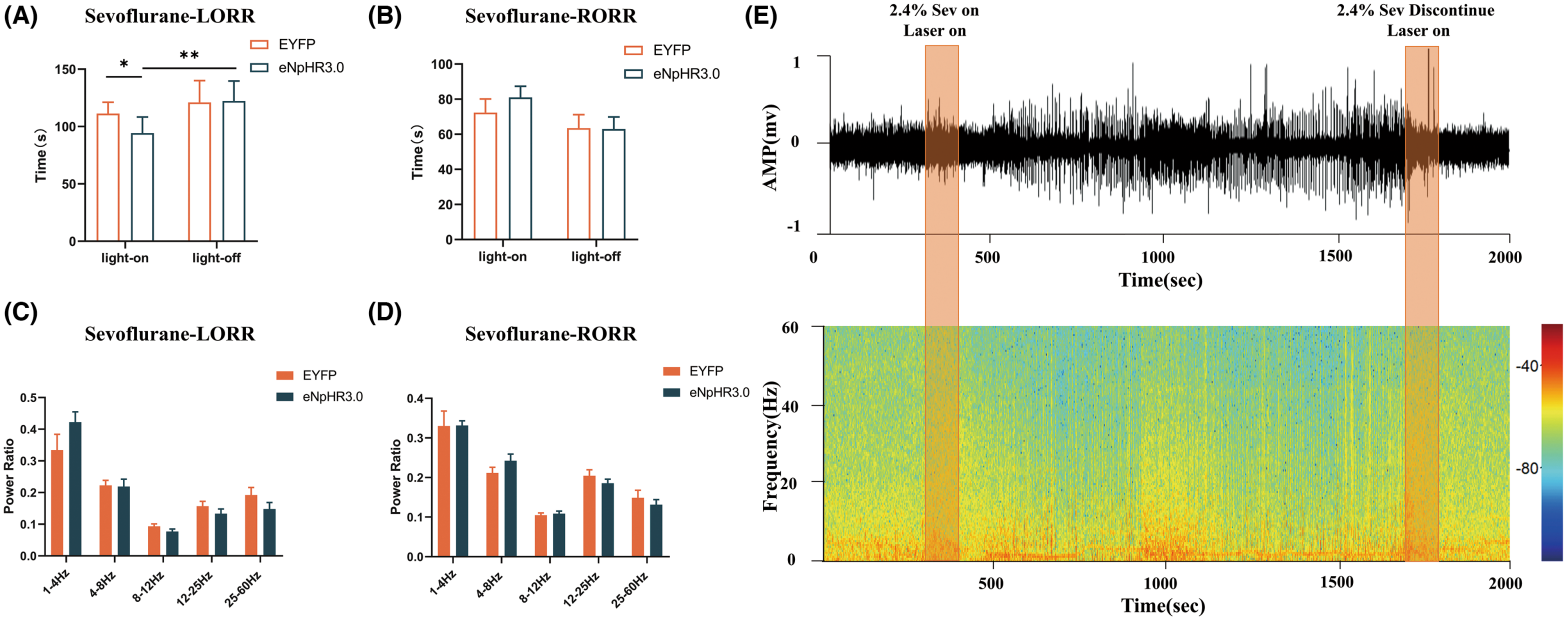
Behavioral and EEG alterations of photoinhibited PBN^vglut2^‐EA under sevoflurane anesthesia. (A) The LORR time was significantly reduced after photoinhibition of PBN^vglut2^‐EA (eNpHR3.0‐light‐on: 94.25 s ± 4.969 s vs. EYFP‐light‐on: 111.3 s ± 3.529 s, *p =* 0.0145, eNpHR3.0‐light‐on vs. eNpHR3.0‐ light‐off: 122.3 s ± 6.151 s, *p =* 0.0033). (B) Photoinhibition of PBN^vglut2^‐EA showed no significant changes in RORR time. (C) Photoinhibition of PBN^vglut2^‐EA during sevoflurane anesthesia induction did not cause any significant changes in any of the EEG frequency bands. (D) Photoinhibition of PBN^vglut2^‐EA during sevoflurane anesthesia recovery did not cause any significant changes in any of the EEG frequency bands. (E) Photoinhibited PBN^vglut2^‐EA EEG spectra and heatmap. The results are presented as the mean ± SEM, *n* = 8 in each group, *p < 0.05, **p < 0.01.

To specifically inhibit the PBN^vglut2^‐EA terminals, an inhibitory optogenetic virus (NpHR) was injected into the PBN, followed by the implantation of optical fibers into the EA of vGLUT2‐Cre mice. Photoinhibition (589 nm, 5 mW, 5 ms pulse, 20 Hz) of the PBN^vglut2^‐EA during anesthesia induction reduced the LORR time (eNpHR3.0 light on: 94.25 s ± 4.969 s vs. EYFP light on: 111.3 s ± 3.529 s, *p =* 0.0145, eNpHR3.0 light on 94.25 s ± 4.969 s vs. eNpHR3.0 light off: 122.3 s ± 6.151 s, *p =* 0.0033, *n* = 8 per group) (Figure 4A). The RORR time showed no significant differences when photoinhibition was administered (Figure 4B).

EEG changes were recorded during optogenetic inhibition, and there were no significant differential changes during either the LORR or RORR time (Figure 4C–E). These results suggest that the induction time of sevoflurane anesthesia can be shortened after photoinhibition of the PBN^vglut2^‐EA.

### 3.5 Sex differences in PBN^vglut2^ ‐EA neuronal activity during sevoflurane anesthesia

**FIGURE 5:**
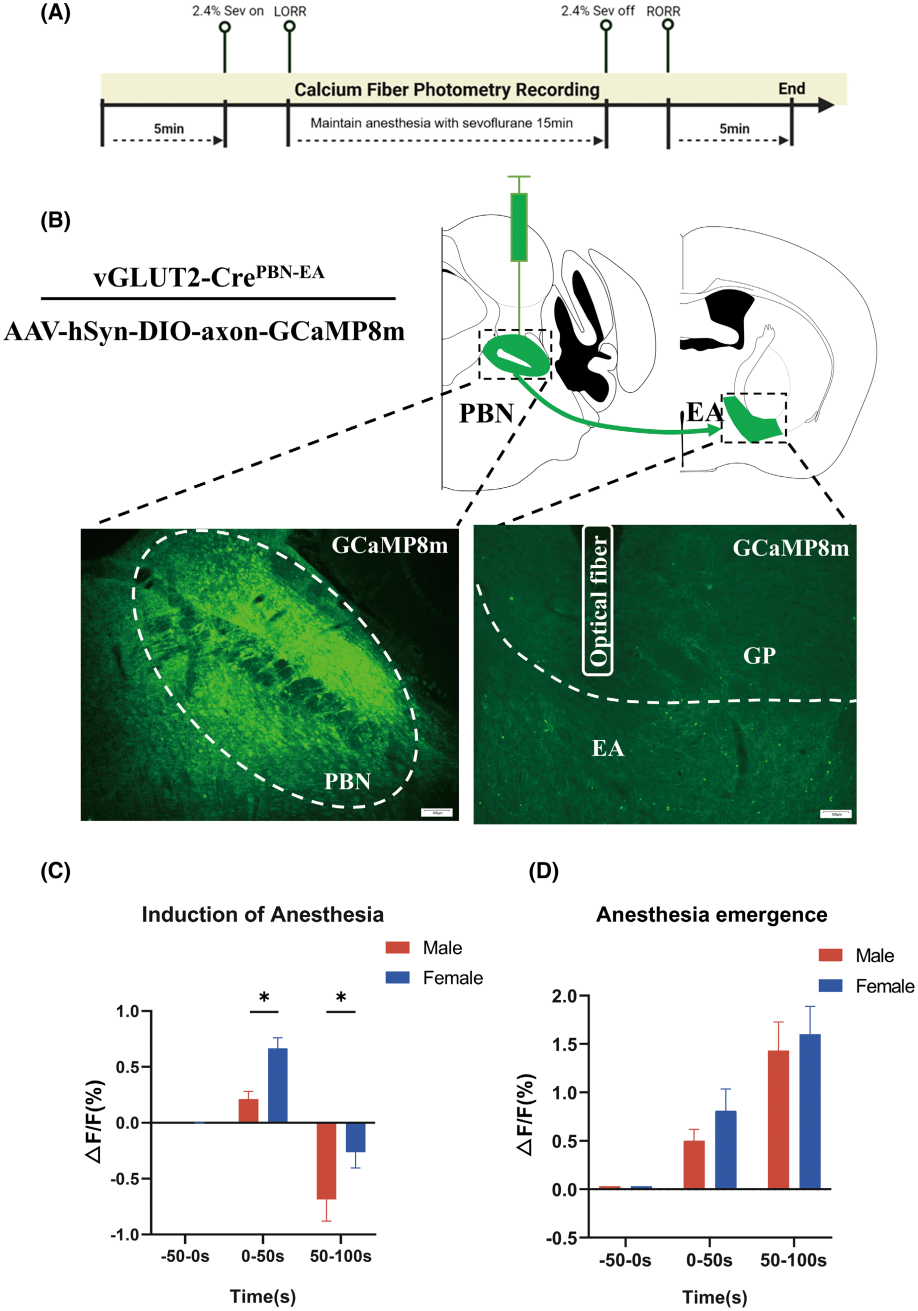
Sex‐specific calcium activity of PBN^vglut2^‐EA terminals during sevoflurane anesthesia. (A) Schematic of the in vivo fiber photometry recording procedure (Created with BioRender.com). (B) Schematic of GCaMP8m expression in the PBN^vglut2^‐EA and the optical fiber location of a vGLUT2‐Cre mice. Scale bars, 100 μm. (C) During the induction of anesthesia, the activation of the PBN^vglut2^‐EA in phase II was lower in males than in females (males: 0.2119 ± 0.06901 vs. females: 0.6659 ± 0.09575, *p* = 0.0147) and was significantly decreased in phase III (males: −0.6856 ± 0.1939 vs. females: −0.2641 ± 0.1398, *p* = 0.0259). (D) There was no significant sex difference in PBN^vglut2^‐EA calcium activity signal during recovery from sevoflurane anesthesia. The results are presented as the mean ± SEM, *n* = 9 in each group, *p < 0.05.

In this study, we included the female group for observation and employed the same fiber photometry approach for comparative analysis with males (Figure 5A). The virus was injected into the PBN of vGLUT2‐Cre mice and an optical fiber was positioned above the EA for measurement of PBN‐EA glutamatergic terminals calcium activity (Figure 5B). During the induction of anesthesia, males showed significantly lower calcium activity expression in phase II than females (males: 0.2119 ± 0.06901 vs. females: 0.6659 ± 0.09575, *p* = 0.0147, *n* = 9 per group). There was a decreasing trend in calcium activity expression as they entered early phase III of anesthesia, with males showing a significant decrease compared to females (males: −0.6856 ± 0.1939 vs. females: −0.2641 ± 0.1398, *p* = 0.0259, *n* = 9 per group) (Figure 5C). During the anesthesia recovery period, there were no significant differences in calcium activity expression observed between males and females (Figure 5D). These results suggest that there are sex differences in PBN^vglut2^‐EA activity during sevoflurane anesthesia, with higher calcium activity in females than in males during the induction of anesthesia.

### 3.6 Sex‐specific changes in consciousness associated with optical stimulation of PBN^vglut2^ ‐EA during sevoflurane anesthesia

**FIGURE 6:**
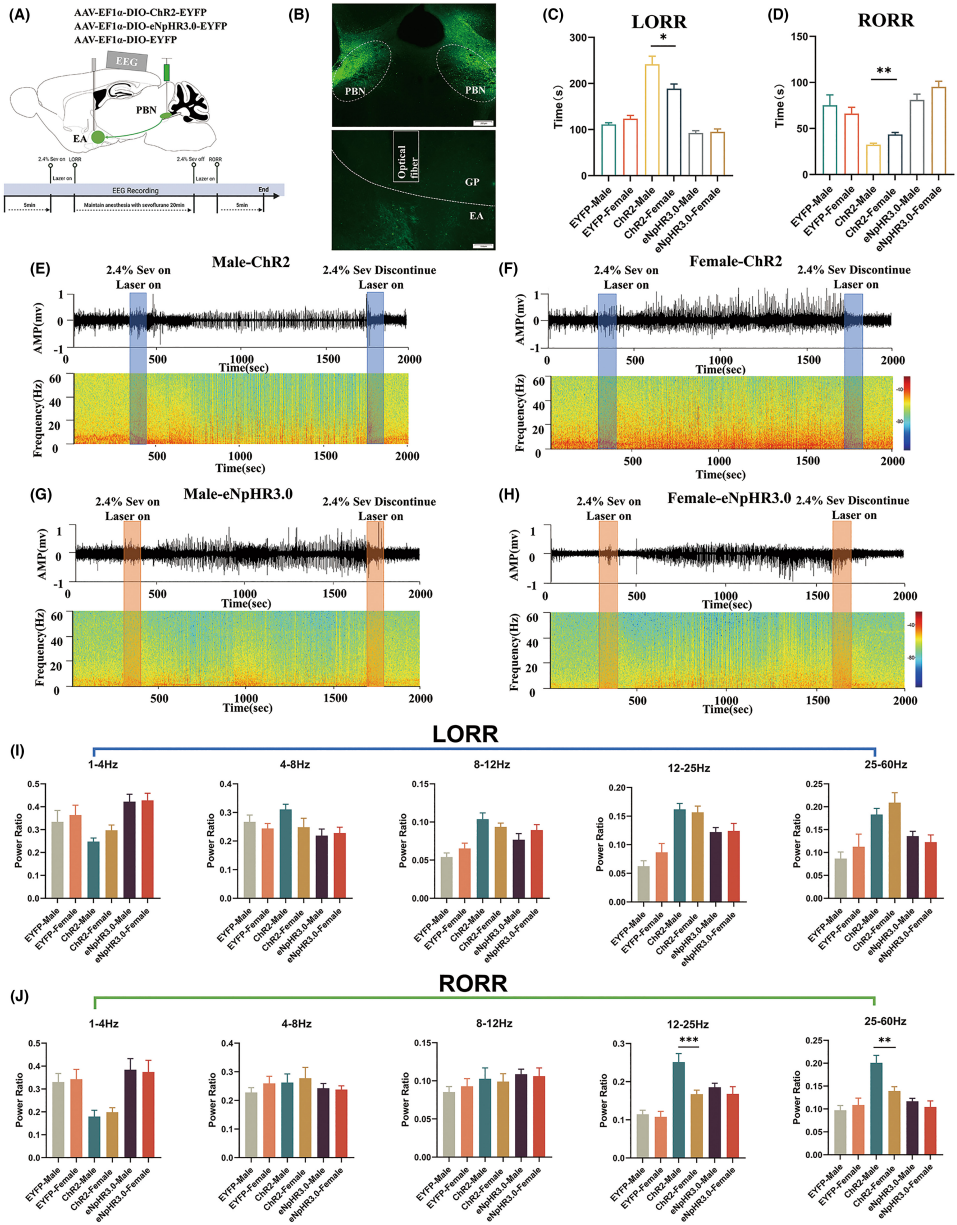
Sex‐specific behavioral and EEG changes following optogenetic stimulation of the PBN^vglut2^‐EA during sevoflurane anesthesia. (A) Schematic of virus injection in PBN^vglut2^‐EA and optogenetic stimulation procedure (Created with BioRender.com). (B) Schematic of virus expression in PBN and the location of the optical fiber in vGLUT2‐Cre mice. (C) Photoactivated PBN^vglut2^‐EA during sevoflurane anesthesia caused the LORR time in males to significantly increase compared to females (males: 241.9 s ± 17.43 s vs. females: 188.8 s ± 9.987 s, *p =* 0.0159); (D) Photoactivated PBN^vglut2^‐EA during sevoflurane anesthesia caused the RORR time in males to significantly decrease compared to females (males: 32.38 s ± 1.66 s vs. females: 43.50 s ± 2.175 s, *p =* 0.0012); (E) EEG spectra and heatmap of photoactivated PBN^vglut2^‐EA in males; (F) EEG spectra and heatmap of photoactivated PBN^vglut2^‐EA in females; (G) EEG spectra and heatmap of photoinhibited PBN^vglut2^‐EA in males; (H) EEG spectra and heatmap of photoinhibited PBN^vglut2^‐EA in females; (I) None of the EEG frequency bands showed significant changes during sevoflurane anesthesia; (J) Optical activation caused the relative power of β oscillations to be significantly higher in males 0.2514 ± 0.02232 vs. females: 0.1672 ± 0.01075, *p =* 0.0010, and the relative power of γ oscillations was significantly increased in males compared to females (males: 0.2009 ± 0.01632 vs. females: 0.1395 ± 0.00917, *p =* 0.0029). The results are presented as the mean ± SEM, *n* = 8 in each group, *p < 0.05, **p < 0.01, ***p < 0.001.

To determine the modulation characteristics of sex differences in PBN^vglut2^‐EA under sevoflurane anesthesia, we utilized optical stimulation for observation. The same optogenetic approach procedure was employed (Figure 6A). We injected excitatory optogenetic virus (ChR2)/inhibitory optogenetic virus (NpHR) into the bilateral PBN of vGLUT2‐Cre mice and positioned optical fibers above the EA (Figure 6B) for photostimulation of PBN‐EA glutamatergic terminals. During the induction of sevoflurane anesthesia, there were significant sex differences in the LORR after photoactivation of the PBN^vglut2^‐EA in vGLUT2‐Cre mice. Males showed significantly prolonged LORR times compared to females (males: 241.9 s ± 17.43 s vs. females: 188.8 s ± 9.987 s, *p =* 0.0159, *n* = 8 per group) (Figure 6C), whereas no significant differences were observed during photoinhibition. After cessation of continuous sevoflurane inhalation and photoactivation of the PBN^vglut2^‐EA, the RORR time was significantly shorter in males than in females (males: 32.38 s ± 1.66 s vs. females: 43.50 s ± 2.175 s, *p =* 0.0012, *n* = 8 per group) (Figure 6D). No significant differences were observed in the photoinhibited group.

The EEG changes were observed in both the female and male groups during the entire procedure (Figure 6E–H). During sevoflurane anesthesia, EEG recordings during the LORR showed no significant differences in PBN^vglut2^‐EA activity between the sexes. However, during the RORR, males exhibited significantly higher relative power of β oscillations compared to females (males: 0.2514 ± 0.02232 vs. females: 0.1672 ± 0.01075, *p =* 0.0010, *n* = 8 per group). Additionally, γ oscillations were significantly higher in males than in females (males: 0.2009 ± 0.01632 vs. females: 0.1395 ± 0.00917, *p =* 0.0029, *n* = 8 per group) (Figure 6J). The findings indicate that the activation of PBN^vglut2^‐EA in males may reduce the sensitivity to sevoflurane anesthesia and is associated with alterations in EEG activity.

## 4 DISCUSSION

In this study, we revealed the role of PBN^vglut2^‐EA in the regulation of the general anesthesia process. Specifically, (1) we confirmed that EA is structurally connected to the PBN by receiving afferent neuronal projections from PBN_vglut2_ neurons and that its neural activity changes after sevoflurane anesthesia. (2) We found that optogenetic activation of the PBN^vglut2^‐EA delayed the induction time of sevoflurane anesthesia and accelerated emergence, accompanied by EEG changes. Additionally, we observed changes in the anesthetic behavioral response to photoinhibition of the PBN^vglut2^‐EA. More importantly, (3) we demonstrated sex‐specific changes that occur during the induction of sevoflurane anesthesia. Optogenetic activation of the PBN^vglut2^‐EA decreased sensitivity to sevoflurane in males but not in females and was accompanied by characteristic changes in the EEG.

The parabrachial nucleus is essential for sustaining wakefulness by regulating glutamatergic neurons during general anesthesia.^14^, ^15^ Studies have shown that the PBN is anatomically and functionally related to the BNST and CeA.^24^, ^25^, ^26^, ^27^ Using anterograde tracer, we successfully mapped the projection of PBN_vglut2_ to the EA. Then, we confirmed the function of the nucleus EA in sevoflurane anesthesia through c‐Fos immunofluorescence and monitored alterations of the PBN^vglut2^‐EA connection during sevoflurane anesthesia using calcium photometry fiber recordings. These results indicate a transient increase in calcium activity of the PBN^vglut2^‐EA terminals during the induction of anesthesia. Evidence has shown that the PBN is a multisensory relay station involved in the transmission of somatosensory signals from the spinal cord and cranial nerves.^28^ Additionally, the CeA and BNST are critical in stressor‐related regulation (e.g., anxiety, fear, stress, drug stimulation, etc.).^29^, ^30^ Therefore, the transient increase in calcium activity of the PBN^vglut2^‐EA terminals may be caused by the stimulation of the mice with the specific odor of sevoflurane itself. We previously reported that the calcium activity of the PBN showed a significant peak during general anesthesia recovery.^14^ In the study, we also observed that the cessation of inhalation of sevoflurane was shortly followed by peaks in calcium activity during the anesthesia emergence phase. Thus, the PBN^vglut2^‐EA may play a role in maintaining arousal and regulating behavior during anesthesia.

To further explore the regulatory characteristics of PBN^vglut2^‐EA in sevoflurane anesthesia, we used an optogenetic approach in combination with EEG. The photoactivation of PBN^vglut2^‐EA resulted in an extended induction duration and a reduced recovery time from anesthesia. In previous studies, sevoflurane, a commonly used anesthetic, has been shown to decrease calcium‐dependent glutamate release in a dose‐dependent manner.^31^ However, activation of PBN^vglut2^‐EA reverses the anesthetic effects of sevoflurane. In general anesthesia, EEG recordings usually show an increase in the power of δ oscillations and a decrease in that of γ oscillations.^32^, ^33^, ^34^ In addition, volatile anesthetics cause a reduction in the power of β oscillations.^35^, ^36^ The photoactivation of PBN^vglut2^‐EA in our study triggered cortical activation, causing a decline in the relative power of δ oscillations and a simultaneous increase in the power of β and γ oscillations. It has been found that optogenetic activation of GABAergic neurons in the BNST can lead to an immediate transition from NREM sleep to wakefulness,^21^ accompanied by an increase in the power of γ oscillations.^37^, ^38^, ^39^ Interestingly, high‐frequency oscillations of 110–160 Hz activity have been observed in the BNST and CeA during the sleep–wake cycle, and this may be due to interactions between GABAergic neurons.^40^, ^41^ Furthermore, β oscillations are associated with GABAergic activity,^42^ which is relevant to the ratio of GABA to glutamate under general anesthesia.^34^ Hence, we propose that activating PBN^vglut2^‐EA increases glutamate release, affecting GABAergic neuron activity in the EA region. This, in turn, regulates cortical arousal and associated EEG patterns. However, this hypothesis requires further investigation in the future.

Loss of consciousness during general anesthesia is caused by fragmentation of information transfer between cortical areas.^43^ General anesthetic agents can interfere with the connections between the subcortical arousal nuclei, thereby reducing the cortical ability to transmit and integrate information.^44^, ^45^ In this process, the LORR may be attributed to alterations in oscillation patterns, as a consequence of disrupted information transfer and integration between cortical regions.^46^ It has been found that continuous δ oscillations can lead to LORR.^47^ During the induction of anesthesia with sevoflurane, photoinhibition of the PBN^vglut2^‐EA only resulted in a reduction of the induction time. Nevertheless, we observed a positive trend in the increase of δ oscillations during the process. Studies indicate that the PBN is linked to other arousal‐promoting nuclei, including the prefrontal cortex, basal forebrain, lateral hypothalamus, and thalamus.^11^, ^14^, ^48^ These nuclei are believed to be involved in the regulation of arousal.^11^, ^14^, ^48^ Therefore, it can be inferred that photoinhibition of PBN^vglut2^‐EA results in the suppression of its ability to promote arousal. This process may result in the activation of a compensatory feedback mechanism that strengthens the functional links between the PBN and other arousal promoting nuclei. Consequently, this counteracts the inhibition and maintains the oscillations in a stable state.

A new study showed that subcortical areas display sex dimorphic activity during anesthesia, and such differences in anesthetic sensitivity are mostly attributed to the effects of sex hormones.^49^ Notably, the PBN contains a large number of glutamatergic neurons and a dense distribution of estrogen receptors (ER) in the caudal‐medial and lateral PBN.^50^ The inhibitory effect of estrogen on the PBN has been found to be mediated by GABA receptors and may cause a negative effect on sleep.^51^, ^52^ In our study, we also examined the sex‐related changes in PBN^vglut2^‐EA regulation under sevoflurane anesthesia. We found a significantly high level of calcium activity in females compared to males during the induction of anesthesia. This result may be caused by estrogen regulation, which can enhance glutamatergic neurotransmission by activating NMDA receptors.^53^ Studies have revealed variations in the ER subtypes that mediate neuroexcitatory transmission in the hippocampus, with presynaptic glutamatergic neurotransmission regulated by ERβ in females and ERα in males.^54^ In another study, ERα‐mGluR1 and mGluR1‐IP3R complexes were found in the hippocampus of both sexes, but only regulated by 17β‐estradiol in females.^55^ Furthermore, prolonged exposure to high concentrations of sevoflurane significantly reduces levels of glutamine, glutamate, aspartate, and proline and estrogen regulation can slow the rate of decrease in glutamate levels.^56^

Additionally, we found sex‐dependent differences in sevoflurane anesthesia with males displaying greater sensitivity than females.^57^ Studies have demonstrated that specific inhibition of ERα in the medial preoptic area altered the sensitivity and depth of anesthesia, delayed the time of induction of anesthesia, and reduced the time of awakening only in males.^57^ These findings may clarify why males demonstrate an extended induction time and reduced emergence time following the specific activation of PBN^vglut2^‐EA at an equivalent sevoflurane concentration. Moreover, a recent study showed that female mice are more resistant to volatile anesthetics than males,^49^ which is consistent with our findings. As mentioned above, the power of β oscillations induced by volatile anesthetics is reduced and it is related to GABA neurotransmission regulation.^35^, ^42^ Our results showed that male mice increased β and γ oscillations during the recovery of sevoflurane anesthesia in response to activation of the PBN^vglut2^‐EA. Estrogen is known to inhibit neurotransmission in the PBN by interacting with GABA_A_ receptors.^52^ In addition, estrogen may provide neuroprotection by inhibiting glutamate‐induced neuroexcitotoxicity.^58^ Thus, we suppose that females could maintain homeostasis by protecting the ER following optogenetic stimulation of the glutamatergic pathway. In contrast, males exhibit a more pronounced activation attributed to a deficiency in estrogenic regulation.

Several limitations of this study should be considered. The modulation of PBN^vglut2^‐EA under sevoflurane anesthesia has been explored. Further research is required to investigate the neuronal types that receive input signals in the EA and the relevant neural circuits involved in general anesthesia. Sex differences in the effects of PBN^vglut2^‐EA on sevoflurane‐induced changes in consciousness were observed. Further investigation is necessary to determine whether this is attributable to estrogen regulation. Future research will focus on addressing these limitations.

Overall, our study indicates that PBN^vglut2^‐EA is involved in the modulation of awakening under sevoflurane anesthesia. Meanwhile, PBN^vglut2^‐EA may exhibit sex‐specific activities under general anesthesia. The activation of PBN^vglut2^‐EA reduces sensitivity to sevoflurane in males, resulting in enhanced arousal states and EEG changes.

## AUTHOR CONTRIBUTIONS

Weifeng Yu, Tian Yu, Yan Yan, Yingfu Jiao, and Hongxin Dong collaborated on the study design and manuscript writing. Yan Yan, Enpeng Liang, Xiaolu Lei, Nan Zhang, and Shimao Xu performed calcium fiber photometry and optogenetic experiments. Yan Yan, Lin Zhang, Jiedong Wang, and Chengdong Yuan conducted the sample collection and immunofluorescence staining. Yan Yan, Tianyuan Luo, Jie Yuan, and Hao Yang were responsible for data processing. All authors contributed to the article and approved the submitted version.

## CONFLICT OF INTEREST STATEMENT

The authors declare that they have no known competing financial interests or personal relationships that could have appeared to influence the work reported in this paper.

## Data Availability

All data reported in this study are available from the corresponding author upon reasonable request.
